# A Model of Intervention and Implementation of Quality Building and Quality Control in Childcare Centers to Strengthen the Mental Health and Development of 1-3–Year Olds: Protocol for a Randomized Controlled Trial of Thrive by Three

**DOI:** 10.2196/17726

**Published:** 2020-10-13

**Authors:** Ratib Lekhal, May Britt Drugli, Turid Suzanne Berg-Nielsen, Elisabet Solheim Buøen

**Affiliations:** 1 Department of Communication and Culture Norwegian Business School Oslo Norway; 2 Department of Education University of Oslo Oslo Norway; 3 The Regional Centre for Child and Youth Mental Health and Child Welfare - Central Norway Norwegian University of Science and Technology (NTNU) Trondheim Norway; 4 Inland Norway University of Applied Sciences Centre of the Study of Educational Practice Lillehammer Norway; 5 Regional Centre for Child and Adolescent Mental Health Eastern and Southern Norway Oslo Norway

**Keywords:** childcare quality, children’s mental health, intervention, RCT, CLASS, well-being, pediatrics, mental health, toddler, children

## Abstract

**Background:**

Universal, high-quality childcare offers a unique opportunity to prevent developmental trajectories leading to mental health problems. Yet, growing evidence has shown that the process quality of Norwegian childcare centers varies considerably, and that research-based models for quality building are significantly lacking.

**Objective:**

To examine whether a model for quality building in childcare centers, Thrive by Three, increases the quality of child–caregiver interactions, and promotes child development, well-being, and mental health.

**Methods:**

The Thrive by Three study is a clustered randomized controlled trial involving 187 toddler groups in childcare centers across 7 municipalities within southern and central Norway. Each center is randomly allocated to the intervention or wait-list control group. Data are collected at 4 points: preintervention (T1), midway (T2), postintervention (T3), and 1-year postintervention (T4). Primary outcomes are changes in childcare quality measured by the Classroom Assessment Scoring System toddler version (CLASS), Student–Teacher Relationship Scale, Short Form (STRS-SF), and Life in Early Childhood Programs (LECP), as well as child development and mental health measured by The Brief Infant Toddler Social and Emotional Assessment (BITSEA, parent and teacher report), the Caregiver–Teacher Report Form (C-TRF), and Child Behavior Checklist (parent report) from the Achenbach System of Empirically Based Assessment (ASEBA) from 1.5 to 5 years, and child well-being measured by the Leiden Inventory for Child’s Well-Being in Day Care (LICW-D). Secondary outcomes are child cortisol levels, assessed in a subsample of 372 children.

**Results:**

As of August 2020, a total of 1531 children and 769 staff from 187 toddler groups were recruited. Because of turnover, the recruitment of staff will be ongoing until August 2020. As of January 2020, the intervention group has been working with Thrive by Three for 1.5 years. Data at T1, T2, and T3 from both the intervention and control groups have been completed and T4 will be completed in August 2020.

**Conclusions:**

This study makes an important contribution to the field of quality building in childcare centers. The results will provide greater insight into how high quality can be obtained and the effects of high-quality early childcare on child mental health. This in turn will be significant for policymakers and to the Norwegian society at large.

**Trial Registration:**

ClinicalTrials.gov NCT03879733; https://clinicaltrials.gov/ct2/show/NCT03879733 and Norwegian Research Council 260624/H10; https://prosjektbanken.forskningsradet.no/#/project/NFR/260624/Sprak=en

**International Registered Report Identifier (IRRID):**

DERR1-10.2196/17726

## Introduction

### Background

Mounting evidence supports the notion that high-quality childcare centers promote children’s mental health [1,2]. High-quality childcare protects and promotes the underpinnings of mental health in a phase of life when brain plasticity is most pronounced [3,4]; hence, it may prevent the onset of deviant developmental trajectories in toddlers and compensate for insufficient resources at home [5,6]. Evidence also suggests that centers of poor quality can have detrimental effects on children’s development [7,8]. Because more than 80% of 1 and 2 year olds in Norway attend childcare, and stay there for long hours [9], interventions targeting caregiver–child interactions may prove an important avenue toward universal prevention of mental health problems.

Childcare quality includes elements of both structure (eg, number of staff, staff training and education, group size, child-to-caregiver ratio) and process (eg, caregiver–child relationship, parental involvement) [10]. *Process quality* in terms of warm, sensitive, and stimulating relationships between caregivers and young children [11] is arguably the most important ingredient of quality as it has long-term effects on children’s mental health, well-being, and development [12]. Importantly, high process quality seems to be particularly important for children at risk [13].

New evidence shows that quality varies substantially between centers [14,15], and preliminary findings indicate that, by international comparison, quality in Norway is only moderately high [15]. Norway has failed to implement a minimum level of training for all childcare staff. As much as 60% of childcare staff have no formal qualifications on bachelor level for working with children [16]. Even some educated childcare teachers report lack of competence in taking care of children under the age of 3 [17]. This means that many toddlers spend most of their time interacting with staff with minimal or insufficient training. The need to enhance competence among childcare staff working with the youngest children is urgent. Moreover, a standardized and systematic procedure for measuring and monitoring process quality in childcare centers is lacking in Norway. This means that, unless a childcare center has participated in research, the level of quality that childcare centers provide to young children is largely unknown.

Few of the existing interventions for preventive quality improvement in childcare specifically focus on the youngest children. Furthermore, most interventions have been narrow in their scope, targeting only single topics such as conduct problems [18], anxiety [19], or language problems [20], thereby limiting their effect on children’s mental health. We will therefore break new grounds by conducting a cluster randomized controlled trial (RCT) of a newly developed comprehensive intervention for promoting mental health among toddlers through quality-enhancement in childcare, the *Thrive by Three* [21-23].

Thrive by Three is grounded in transactional models for development [24,25] and research showing that interactions between young children and caregivers are primary mechanisms of mental health, child development, and learning [26-29]. Thrive by Three is culturally sensitive, and is universally preventive in the sense that it includes all 1-3 year olds attending childcare. The intervention consists of 3 core elements: (1) quality building through competence enhancement for all staff regardless of level of formal education, (2) a model for peer-driven quality control and tailored supervision at classroom level in the participating centers, and (3) supervision of supervisors and mentors. Thrive by Three is modeled after the *Thrive by Five*, *Seeds to Success*, and *Early Achievers* quality-building and quality control measures, broadly implemented in childcare centers in the state of Washington in the United States [21-23] and piloted in a large Norwegian community sample [30].

This study has 2 primary aims: to determine the extent to which Thrive by Three (1) improves the quality of caregiver–child interactions and (2) strengthens children’s mental health, and social and cognitive development, after the intervention and at a 12-month follow-up. The secondary aims consist of determining the extent to which the intervention effects are moderated by (1) the implementation outcomes (fidelity and acceptability) and (2) child and family characteristics such as temperament, ethnicity, and socioeconomic background. In a subsample, we will also address (1) the characteristics of children with especially high cortisol levels during transition to childcare and (2) whether children in childcare units receiving the intervention have lower cortisol levels compared with controls. In another subsample we will qualitatively investigate parents’ and caregivers’ experience with the intervention.

### Pilot Study

The Thrive by Three intervention was piloted in a large community sample in 2 cohorts of 25/49 public (51%) and 24/49 private (49%) childcare centers from August 2016 to June 2018. A total of 49 centers and 243 groups of children aged 1-5 participated. The design was a simple pre–post design. The childcare centers were observed with Classroom Assessment Scoring System (CLASS; toddler and Pre-K), before the intervention started (pre) and after 10 months (post). Childcare staff filled out electronic questionnaires on background factors of the centers, the childcare classrooms, and themselves (ethnicity, education, staff turnover, attitudes toward the intervention). The pilot intervention differed from the current version of Thrive by Three intervention in 2 ways:

1. The written material given to the staff was less comprehensive in the pilot and parents were not given any written material.
2. The staff supervisors did not receive supervision from mentors.

For toddler groups (ages 1-3) we found a small but significant increase in quality from pre to post in the Emotional and behavioral support domain (mean 5.97 [SD 0.41] vs mean 6.08 [SD 0.38]; *t*_103_=–2.45; *P*=.02) but not in the Engaged support for learning domain (mean 3.24 [SD 0.76] vs mean 3.44 [0.77]; *t*_103_=–1,86; *P*=.07). For the Pre-k groups (ages 3-5), there was a significant increase in quality from pre to post measures across all 3 domains: Emotional support (mean 6.06 [SD 0.56] vs mean 6.20 [SD 0.43]; *t*_127_=–2.56; *P*=.01), Classroom organization (mean 5.27 [SD 0.76] vs mean 5.55 [SD 0.66]; *t*_127_=–3.38; *P*=.001), and Instructional support (mean 2.67 [SD 0.84] vs mean 3.02 [SD 0.98]; *t*_127_=–3.40; *P*=.001). Moreover, qualitative interviews with center leaders, head teachers, and staff from the first cohort (N=53) revealed great satisfaction with the quality-building framework and the holistic approach to quality building. Overall, they reported feeling more competent in their day-to-day interactions with the children and highlighted that the intervention had given all staff on different levels shared knowledge and a shared language to talk about quality with colleagues. There were, however, also several challenges. Most importantly, staff needed more time than predicted to fully comprehend the CLASS framework and content of the specific dimensions. The supervisors did not receive enough support and reported lacking a manual/written material to help structure the supervision throughout the intervention period.

## Methods

### Study Design

This study is a clustered RCT delivered in childcare centers with toddler groups in 2 regions in Norway: southern and central. We selected a random sample using SPSS (IBM). The stages of enrollment, intervention, and assessment are presented in Table 1. A more detailed display of the different assessment time points is presented in Table 2.

**Table 1:** The stages of enrollment, intervention, and assessment of Thrive by Three.

Research stage		Study period						
		Spring 2017	Fall 2017	Spring 2018	Fall 2018	Spring 2019	Fall 2019	Spring 2020
Enrollment								
Municipalities		x						
**Informed consent**								
	Staff	x	x					
	Children		x	x				
Allocation			x					
**Intervention**								
	Intervention group				x	x		
	Control group						x	x
**Assessment**								
	Primary and secondary outcomes				Pre	Post		1-year follow up

**Table 2:** Overview of assessment and informants at different time points.

Assessment and informants		Time point			
		T1 (September 2018)	T2 (January 2019)	T3 (June 2019)	T4
**Questionnaires**					
	Childcare staff about their job	x		x	x
	Childcare staff about the child	x		x	x
	Parents about themselves and child	x		x	x
CLASS^a^ observations		x	x	x	x
Cortisol measurement (300 children)		x	x	x	

^a^CLASS: Classroom Assessment Scoring System.

### Eligibility Criteria

Childcare settings with toddler groups in the Oslo region and Central Norway are eligible for participation. The participating children must be aged between 12 and 35 months. Childcare center eligibility is determined by region of the country and municipality. The primary study sites are in southeastern and central parts of Norway. The recruitment period started in February 2017 and lasted until August 2018. First, municipalities were recruited during the spring of 2017. Then, childcare centers within each municipality were recruited during spring and fall of 2017. Childcare staff consented during the fall of 2017. Children were recruited from fall of 2017 until August 2018. The long recruitment period of children is due to the fact that children under the age of 2 typically enter childcare centers for the first time in August, the year they turn 1. The lists of eligible children were therefore not complete until August 2018. Because of a high staff turnover, new staff members are invited to consent to participate throughout the year.

The intervention is offered during a complete year cycle in the participating centers. The control group is offered the intervention the following year cycle. Childcare quality is measured before, during, and after the intervention (3 time points). Parents and staff fill out questionnaires before and after the intervention. In addition, participating toddler groups and children will be measured 1 year after the intervention to identify possible long-term effects.

Children in the participating toddler groups are recruited via a written invitation to their parents given to them at the childcare center. Childcare staff keep in touch with the parents throughout the study period. The project team has no direct contact with parents. All information is given through the childcare center.

### Inclusion Criteria

All childcare centers in the participating municipalities were allowed to participate as long as they had at least one toddler group, or groups with children aged 12–36 months. After the municipalities had consented to participate in the study, childcare centers within each municipality were recruited. Childcare centers volunteered to participate. All parents of children in the toddler groups of the participating childcare centers were invited to participate.

### Exclusion Criteria

Childcare centers with no children under the age of 36 months were not eligible for the study.

### Intervention Care and Comparison

#### Intervention

Thrive by Three is a 10-month intervention, aimed at increasing the quality of Norwegian childcare centers. It is grounded in transactional models for development [24,25] and research showing that interactions between young children and caregivers are a primary mechanism of mental health, child development, and learning [26-29].

Thrive by Three offers a standardized measurement of quality 3 times throughout the year through observations conducted by 25 certified observers (from the municipality) in classrooms with children between 1 and 3 years of age using a standardized observations method, CLASS toddler. Following observations, all staff in the intervention group receive feedback on a group level on their score based on the 8 quality dimensions of CLASS, showing strengths and weaknesses of interaction. Staff receive systematic guidance by their head teacher on a monthly basis based on the quality measure and an action plan for improvement. The head teachers, in turn, receive supervision from mentors. Staff in each toddler group meet together 10 times with their head teacher during the intervention period, focusing on 1 or 2 CLASS dimensions each time. Between meetings, all employees focus on the present CLASS dimension in their daily work with children.

All childcare employees in the 7 municipalities attend 3 full-day seminars, focusing on research-based knowledge on young children’s mental health and its risk and protective factors, early signs of mental and developmental problems, and crucial aspects of childcare quality. Center leaders and head teachers participate in 2 extra sessions focusing on leadership, feedback, supervision, and how to address concerns about a child to his/her parents when necessary.

In addition, comprehensive written material is provided to parents, employees, head teachers, and mentors. There are 7 booklets for parents about children’s early development and tips to help them support their own child’s development, guidance manuals for mentors, and a 30-page resource booklet for all employees in the centers. Access to the project website is also available to parents and employees at tf3.no.

#### Control

The wait-list control group is offered the intervention 1 year later but is observed at T1, T2, and T3 along with the intervention group. The control group does not receive feedback or guidance following the observations until they receive the intervention 1 year later.

### Procedure

#### Overview

**Figure 1:**
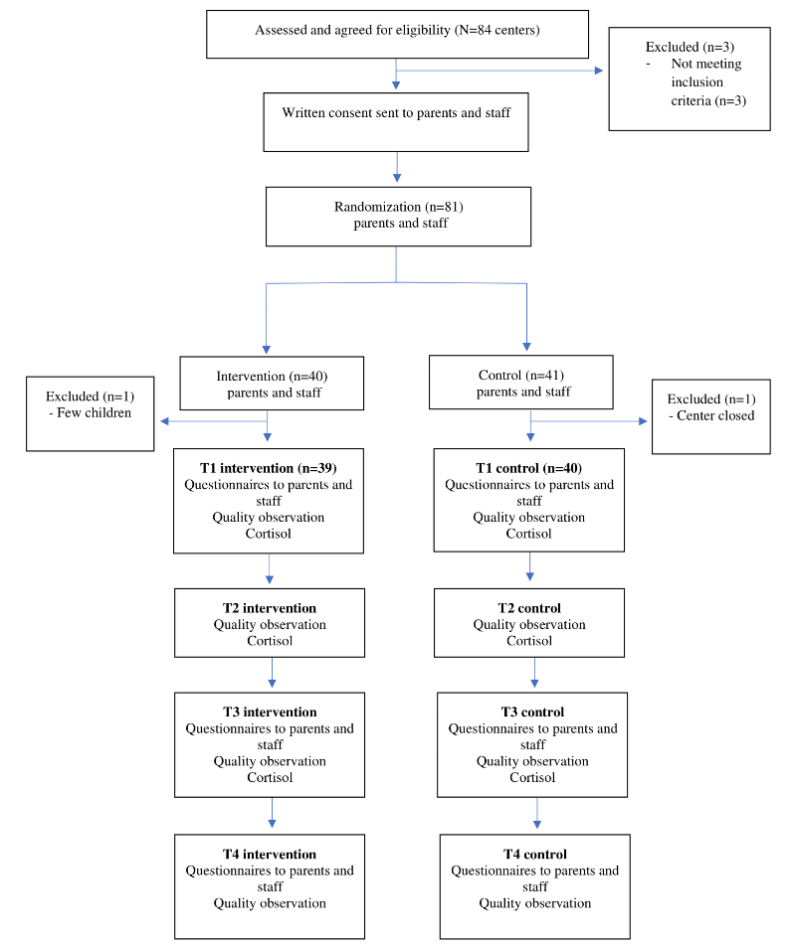
Flowchart of study procedures for Thrive by Three.

Figure 1 presents a graphic representation of the study procedures. Following recruitment, observers and mentors are trained in the childcare quality observation method, CLASS [31]. This is a 2-day training, led by a certified trainer. After training, the Teachstone reliability test is taken. Supervisors get extra training in the method, but do not go through the reliability test to become certified. Each childcare unit is observed by the CLASS-certified observers for 45 minutes, preintervention, midintervention, and postintervention and at 1-year follow-up. The preintervention CLASS observation is used as the basis for supervision and quality enhancement in each unit.

#### Randomization

Childcare centers within each municipality are randomly allocated to the intervention or wait-list control group. Because we could not randomize the units in the same center due to contamination, randomization occurred on the center level, but the intervention is on the unit level. The randomization was stratified based on center size to assure that larger and smaller centers were equally distributed across the control and intervention conditions.

#### Blinding

Because of the nature of the intervention, it is not possible for the participants to be unaware of the intervention condition they are assigned to.

### Outcomes

#### Data Collection

Data are collected at four points: T1 (preintervention), T2 (midway assessment), T3 (postintervention), and T4 (1-year postintervention follow-up). Participants from the intervention and control centers (parents, childcare staff, and group leaders) are sent links to the questionnaire via SMS text message or email. Each child is given unique, confidential identifiers to log-in and answer questions about the child (by the parent/staff). Both parents and 1 selected staff member working with each participating child are asked to fill out the questionnaires about the child. In addition, parents and childcare staff answer questions about themselves, and the staff about aspects of the center they work in. Each unit in the participating centers (both intervention and control centers) is also observed by trained CLASS observers at the following periods: preintervention, midintervention, postintervention, and during a follow-up 1 year later (only the intervention group).

T1 took place in September and October 2018 with CLASS observations in 187 units (both the control and intervention groups, with only the intervention group receiving feedback) and questionnaires to all staff and parents. T2 took place in January 2019 with new CLASS observations but no questionnaires. T3 took place at the end of the intervention (1 childcare year) in May/June 2019 with CLASS observations and questionnaires to all staff and parents. In addition, qualitative interviews will be conducted at T3. At 1-year follow-up after the intervention (T4) the intervention group is observed with CLASS and questionnaires will be filed out by all staff and parents (Textbox 1).

**Study outcomes and measures used for Thrive by Three.**

**Primary outcomes**

- Childcare quality:
  - Classroom Assessment Scoring System (CLASS) toddler version
  - The Student–Teacher Relationship Scale, Short Form (STRS-SF)
  - Life in Early Childhood Programs (LECP)
- Child outcomes:
  - The Brief Infant Toddler Social and Emotional Assessment (BITSEA, parent and teacher report)
  - Caregiver–Teacher Report Form (C-TRF, teacher report)
  - Child Behavior Checklist (CBCL, parent report)
  - Language Development Survey (LDS)
  - Leiden Inventory for Child’s Well-Being in Day Care (LICW-D)
  - Secure Base Safe Haven Observation Unit (SBSHO)

**Secondary outcomes**

- Measurement of child cortisol levels

#### Primary Outcomes

##### Study Hypothesis

The primary outcome for this study is a comparison between centers randomly allocated to the Thrive by Three intervention and wait-list control centers receiving the intervention 12 months later. We hypothesize that Thrive by Three will prove superior to the wait-list control after the 10-month intervention period and at the 12-month follow-up with respect to childcare quality and children’s mental health, development, and well-being.

##### Childcare Quality

The lead teacher in each classroom reports features of the childcare such as type of care, group size, staff-to-child ratio, and characteristics of the child group and staff.

CLASS toddler version [31,32] is used to evaluate process quality. CLASS provides relevant, valid, and reliable information about child–caregiver interactions [31]. Each unit is observed by the CLASS observers for three 15-minute periods (preintervention, midintervention, and postintervention) as well as during a follow-up 1 year later (only the intervention group).

The quality of teacher–child relationships (ie, children’s relationship with teacher) as perceived by the teacher is assessed through 3 dimensions, namely, closeness, conflict, and dependency, using The Student–Teacher Relationship Scale, Short Form (STRS-SF) [33,34], which consists of 15 items.

Life in Early Childhood Programs (LECP) measures the atmosphere, routines, and rhythms of the group [35]. The LECP asks teachers about the degree of control and organization (degree of consistency or routines, if objects or toys are put in the same place), the use of space, contextual traffic (if many people are coming and going), and group density. A higher score on the LECP indicates a higher level of chaos in the group.

##### Child Outcomes

Children’s development and mental health outcomes are determined by serval measures, gathered from both parents and 1 selected staff member working with each participating child.

The Brief Infant Toddler Social and Emotional Assessment (BITSEA, parent and teacher report) [36] is used to map children’s social/emotional/behavior problems and delayed development across 4 domains: externalizing, internalizing, dysregulation, and competence.

Caregiver–Teacher Report Form (C-TRF, teacher report) and Child Behavior Checklist (CBCL, parent report) from the Achenbach System of Empirically Based Assessment (ASEBA) from 1.5 to 5 years [37] are checklists used for mapping emotional difficulties and behavior problems in children. The checklist measures symptoms of anxiety/depression, somatic disorder, withdrawnness, attention difficulties, and aggressive behavior, as well as overarching internalizing and externalizing behaviors. In addition, the Language Development Survey (LDS) is included as part of CBCL for parents to measure which words children use. LDS is answered by mother, father, and teacher for this study.

Leiden Inventory for Child’s Well-Being in Day Care (LICW-D) [38] measures the child’s socioemotional well-being in daycare with 12 items (eg, enjoys attending, feels at ease in the group, not difficult saying goodbye to parents). Mother, father, and teacher answer these questions in this study.

Secure Base Safe Haven Observation Unit (SBSHO) [39] measures children’s secure attachment to their caregiver. Through 20 questions, parents and the child’s key person in the group are asked to evaluate to which degree the child uses them as a secure base.

#### Secondary Outcome: Child Cortisol Levels

Child cortisol levels are assessed in a subsample of 300 children who enter childcare for the first time. The tests are done in the childcare center at 10 am and 3 pm and at home at 6 pm for 2 days at T1 (the average is calculated). At T2, cortisol levels are assessed at 10 am and 3 pm for 2 days in the center and 2 days at home during a weekend (the average for every point in time). At T3, measures are taken in the same way as T1. The collection, storage, and evaluation, including the placement of the material, are approved by Regional Committees for Medical and Health Research [40], reference number 2017/430 [40].

#### Implementation Outcomes

##### Fidelity

Fidelity to the intervention is measured through checklists and self-reporting [41]. Class attendance and attendance in monthly supervision sessions is registered. The supervisors rate their own fidelity of intervention on a 5-point Likert scale (from minimal to high) through questions such as “I helped use the CLASS dimensions and relational quality when the supervisor reflected on practice,” “I focused on how the supervisor could develop as a mentor for their staff.”

##### Acceptability

Acceptability (ie, the users’ satisfaction with various aspects of the intervention) is measured by self-report and focus group interviews with parents and staff at the end of the intervention period.

##### Sustainability

Sustainability is to be measured through semistructured interviews and self-report at the end of the intervention period and at 1-year follow-up [41].

#### Background Variables

##### Child Factors

Serval measures and questions are used to gauge children’s background, including the child’s personal characteristics and aspects of their environment.

The Early Childhood Behaviour Questionnaire, Short Form (ECBQ) [42] measures children’s temperament, including reactive processes that involve emotions, motor and sensory systems, and self-regulation processes that control reactivity.

The Emotionality, Activity, Sociability Temperament Survey for Children (EAS) [43] measures 4 dimensions of the child’s temperament (emotionality, activity, sociability and shyness) on 5 items.

More general characteristics and childcare history are obtained through questionnaire data detailed by parents (eg, number of siblings, preterm, ethnicity, disabilities, when they started kindergarten, how many kindergartens they have attended).

##### Family Factors

Parents report on their own background such as their ethnicity, country of origin, marital status, education, and family socioeconomic status through information about parental income.

##### Childcare Factors

Leaders of the childcare centers report on the organization and size of the childcare center, as well as staff turnover and sick leave. Childcare staff report their gender, ethnicity, age, education, and experience working in childcare. Childcare staff also report on their level of occupational stress and well-being at work, their motivation, and receptiveness to changes in the workplace.

#### Recruitment and Participation

Recruitment and participation data will be reported for available data from baseline.

#### Participant Retention

Contact with childcare staff is maintained by the research group through meetings and emails. Contact with participating families (parents) is maintained mainly by childcare staff. Reminders to fill out questionnaires are sent via email or SMS text messages.

To reduce dropout in the control groups after randomization, childcare centers in the control group receive a gift certificate of 3000NOK (US $336.60) to buy material for their childcare center and are offered the intervention 1 year after the intervention group.

#### Data Management

Observational data are collected by CLASS toddler–certified observers. Questionnaire data are collected and managed by an independent data collection team at the primary sponsor site (Centre for Child and Adolescent Mental Health [RBUP] East and South). The independent data collection team is responsible for monitoring the data as they are collected, as well as checking that the data are consistent and free from errors. Data analysis and cleaning will be performed by study investigators. Data are stored on a secure server at the primary sponsor site during the study and analysis of results. Project staff will have access to the final trial data set. Following the study, data will be anonymized and archived according to Norwegian law.

#### Data Analysis

The study is carried out in toddler groups in childcare centers. Thus, data analyses will be conducted in a multilevel modeling framework to account for nonindependence of the participants at center level.

#### Sample Size and Power

Sample size and power was calculated using SPSS SamplePower (IBM). Based on previous studies [6], we choose a conservative estimate of expected effect of 0.30 (Cohen *d*). With a power of 0.80 and α=.05, we need 352 individuals if the randomization was performed individually. We plan to include data from 16 children from each childcare center. In a similar research [44], the intraclass correlation coefficient (ICC) was reported to be approximately 0.05. However, because several children will be evaluated by the same caregiver, we estimate with an ICC as high as 0.10. The resulting design effect with ICC=0.05 (or 0.10) D=1.75 (or 2.5), and the total required sample size is Nc=352 × 1.75 = 616 (or 352 × 2.5 = 880) individuals. We plan to include 1100 individuals, and 1100/16=69 childcare centers, to account for possibly 20% missing data on individual level, and uncertainty in the effect size and ICC. The planned selection will be in accordance with the Consolidated Standards of Reporting Trials (CONSORT) guidelines for clustered randomized trials [45].

#### Planned Statistical Analysis

To determine the effect of the intervention regression analyses, mixed model analyses or structural equation modeling as appropriate will be conducted, taking into account the clustered or multilevel structure of the data and data related to background factors. Because children are of different ages at study enrollment, some children will stay in intervention classrooms for 2 years, while some for just 1. We will adjust for child age in the analyses to account for these differences in “treatment exposure.” Missing data will be estimated in the models using a well-established technique that allows for the inclusion of all available data and estimation of missing values.

#### Qualitative Analysis

Qualitative analysis of interviews will focus on meaning of texts [46]. Elements from the thematic analysis will therefore be used to analyze the interviews [47]. Analysis will first focus on identifying relevant codes in the text [46]. The next phase will involve sorting the codes into potential overarching themes. The themes will then be reviewed and refined using constant comparative methods to reduce data into essential concepts and relationships. Internal homogeneity and external heterogeneity among themes will be considered in this phase. Three coders will independently categorize the interviews and their coding will be combined [48].

#### Cost

The costs of the intervention will be evaluated by calculating the hours that childcare staff, leaders, CLASS observers, and mentors have donated to the project in relation to the number of toddler groups and children exposed to the intervention. An estimate of per-child cost will be included in the final report to funders, along with additional estimates of costs incurred by the trial research team.

### National Collaboration

This project represents a shared effort and close collaboration between the RBUP (East/South), The Master’s Program for Preschool Leadership at the Norwegian Business School (BI), The National Network for Infant Mental Health in Oslo, Regional Centre for Child and Youth Mental Health and Child Welfare (RKBU) Central Norway at the Norwegian University of Science and Technology (NTNU), and The Psychology Department at NTNU.

### International Collaboration

Professor Robert Pianta (University of Virginia), who led the team that developed CLASS, is a consultant for the research group. The national research team has extensive collaboration with Assistant Professor Gail E. Joseph and her associates. She is the director of the Early Childhood and Family Studies program at the University of Washington and the Principal Investigator and Director of the Childcare Quality and Early Learning Center for Research and Professional Development [49], which is the institute currently investigating effects of the broadband implementation of the quality building and quality control of childcare centers in Washington state. She is also coprincipal investigator and codirector of the National Center for Quality Teaching and Learning.

### Availability of Data and Materials

The data sets or other material used during this study are available from the principal investigator (Elisabet Solheim Buøen) on reasonable request.

### Ethics Approval and Consent to Participate

Ethics approval for the study was given by Regional Committees for Medical and Health Research [40], reference number 2017/430 [49]. We anticipate low risk of harm for participating in the intervention since the work on employee process quality is based on literature and previous research that have shown to be positive for children’s development. In addition, education and guidance for employees is not likely to be harmful for children’s development. Consent was given written by municipalities, childcare centers, and parents. Childcare centers volunteered to participate. All parents of children in the toddler groups of the participating childcare centers were invited to participate and gave a written consent. Consent was given by childcare staff throughout the year.

## Results

### Trial Status

As of August 2020, in total, 1531 children and 769 staff from 187 toddler groups have been recruited. Due to turnover, staff recruitment will be ongoing until August 2020. As of January 2020, the intervention group has been working with Thrive by Three for 1.5 years. Data collection at T1, T2, and T3 from both the intervention and control groups has been completed and T4 will be completed in August 2020. We expect to find an increase in childcare quality and better mental health and development outcomes for children in the intervention group. The reporting of these results will be made in accordance with the CONSORT guidelines [45]. The trial began recruitment in February 2017 and finished in August 2018. Data collection will finish in August 2020.

### Changes to the Protocol

Changes to the project are made in the standard operating procedure. These changes are recorded and maintained by the principal investigator for RBUP East and South. Changes which are merely procedural but may impact the experience of the participants in the study are reported to the Regional Committees for Medical and Health Research ethics for approval.

### Confidentiality

Study participants are provided anonymous ID. A study key with the participants’ name and ID is stored in a separate, encrypted file on an internal server at RBUP East and South.

### Dissemination of Results

Dissemination will be done though scientific publications in well-regarded journals for child mental health and development as well as preventive science journals. Project newsletters and press releases, as well as 2 PhD theses will also be used to disseminate our results. Planned scientific publications include primary outcomes, secondary outcomes, fidelity, and the intervention and implementation. Publication of the data will be in accordance with CONSORT guidelines. The project team has adopted the Vancouver protocol for determination of authorship of scientific publications.

## Discussion

### Protocol Overview

This study is an important contribution to the field regarding the quality of childcare settings in Norway. The results of the study will provide an indication as to whether or not the model for quality building, Thrive by Three, is effective to promote childcare quality within Norwegian childcare toddler groups. The study will also provide information about the effects of Thrive by Three on child development, mental health, and well-being. Finally, the results will provide insight into the implementation of a system for quality building in childcare centers and as such be of great value for Norwegian society, on individual, family, community, and national level.

### Limitations

As with any study, there may be potential limitations and challenges. One of the challenges we anticipate is high dropout from the control group. To counteract this, we are working to motivate the control group by offering them the intervention 1 year later as well as a cash incentive. Another limitation we anticipate is that because the intervention only lasts 1 year, the long-term effects of the intervention are not completely followed up. We do, however, aim to follow the children in the intervention group to see if the positive effects do persist even after the intervention.

### Conclusion

The results of this study will offer a greater understanding of how high-quality childcare can be achieved and the effects of such care on children’s mental health. The study will present significant contributions to the field and present policymakers and the greater Norwegian society with important evidence on quality in childcare.

## Appendix

Multimedia Appendix 1 Original peer-review reports from the funding agency.

## Abbreviations

ASEBA: Achenbach System of Empirically Based Assessment
BI: Norwegian Business School
BITSEA: The Brief Infant Toddler Social and Emotional Assessment
CBCL: Child Behavior Checklist
CLASS: Classroom Assessment Scoring System
CONSORT: Consolidated Standards of Reporting Trials
C-TRF: The Caregiver–Teacher Report Form
EAS: The Emotionality, Activity, Sociability Temperament Survey for Children
ECBQ: The Early Childhood Behaviour Questionnaire, Short Form
ICC: intraclass correlation coefficient
LDS: Language Development Survey
LECP: Life in Early Childhood Programs
LICW-D: Leiden Inventory for Child’s Well-Being in Day Care
NFR: Norwegian Research Council
NTNU: Norwegian University of Science and Technology
RBUP: Centre for Child and Adolescent Mental Health
RKBU: Regional Centre for Child and Youth Mental Health and Child Welfare
SBSHO: Secure Base Safe Haven Observation Unit
STRS-SF: Student–Teacher Relationship Scale, Short Form
