# Supplementary material for: A Model of Intervention and Implementation of Quality Building and Quality Control in Childcare Centers to Strengthen the Mental Health and Development of 1-3–Year Olds: Protocol for a Randomized Controlled Trial of Thrive by Three
Source: JMIR Res Protoc. 2020 Oct 13;9(10):e17726. doi: 10.2196/17726 (PMC7592061; doi:10.2196/17726)
Supplement: Multimedia Appendix 1 [file resprot_v9i10e17726_app1.pdf]

Forskningsrådet

Assessment of grant application submitted to the Research Council of Norway

Grant application

Project number	260624
Project title	Thrive by Three
Project manager	Berg-Nielsen, Turid Suzanne
Project Owner	REGIONSENTER FOR BARN OG UNGES PSYKISKE HELSE, HEL
Programme/Activity	Bedre helse og livskvalitet
Case officer	Torbjørg Øyslebø

Confirmation

By completing and submitting this form, I / we confirm the following (applies for the individual referee or the referee panel):

- I am /We are qualified to assess this application. See Regulations on Impartiality and Confidence in the Research Council of Norway.	Yes
- I/We have read and understood both the criteria I/we have been asked to use for assessing the application and the description of the scale of marks. The scale of marks is to be applied as an absolute scale, i.e. marks are to be determined for each grant application independently and not relative to other applications that the panel/referee is assessing.	Yes
- I/We understand and accept the guidelines for assessing applications for the Research Council of Norway. See Guidelines for referees/panels who assess applications for the Research Council of Norway.	Yes
- I am/We are qualified to conduct this assessment.	Yes

Summary of marks

Criterion	Mark
Relevance relative to the call for proposals	A
Scientific merit	5
The project manager and project group	6
Implementation plan and resource parameters	A
National cooperation	A
International cooperation	A
Dissemination and communication of results	A
User involvement	A
Benefits of the project	A
Overall assessment of the referee/panel	5

Special points to consider	Answer
Ethical perspectives	Yes
Environmental impact	Neutral
Recruitment of women	Significant
Gender balance in the project	Yes
Gender perspectives in the research	Positive
Internationalisation	Significant

Criteria

Relevance relative to the call for proposals

How relevant is the project relative to the call for proposals?

This criterion is used to assess the degree to which the project satisfies the guidelines and stipulations set out in the call for proposals.

The study is highly relevant to the call as it is aiming at prevention of mental health problems and strengthening mental health, development and well-being in very young populations in toddlers between 2-3 years. This is an important aim for improving public health. It is also important to reducing social inequality.
High quality of childcare will be of great value to the Norwegian society and could be used as a model for other countries.

Selected mark : A - Very good
The project fulfils the guidelines in the call for proposals very well.

Scientific merit

How would you rank the project's scientific merit?

This criterion gives an indication of the essential, fundamental aspects of the research project. The scientific merit of a project will be assessed in relation to the following points:
* Originality in the form of scientific innovation and/or the development of new knowledge.
* Whether the research questions, hypotheses and objectives have been clearly and adequately specified.
* The strength of the theoretical approach, operationalisation and use of scientific methods.
* Documented knowledge about the research front.
* The degree to which the scientific basis of the project is realistic.
* The scientific scope in terms of a multi- and interdisciplinary approach, when relevant.

The study is very original in that it is the first such study in this age group in Norway and probably in Europe. The originality is somewhat diminished in that it uses an existing model that has been used in the USA.
The background section is well written and clearly outlines the rationale for the study.The research questions, aims and analyses methods have been described in a very clear and sufficiently detailed manner. The power analysis is solid and so is the data analysis.

There is some lack of consistency between the emphasis in the main objectives set out, the project summary and the main proposal for the research. The main objectives focus on strengthening mental health and improving quality of caregiver behaviour. The secondary objectives relate to moderation of outcomes, fidelity and acceptability, child and family characteristics. There are then questions about cortisol levels and a further question on qualitative investigation of parents and caregivers experience. However the project summary does not mention cortisol, and does refer to cost effectiveness. There is little detail given on cost effectiveness in the main proposal.

With respect to cortisol, the panel wondered about how essential it is to take these measurements at this stage, particularly as the methods include taking samples from very young children during the first day in their new care setting. There has to be a compelling argument for collecting these data, given that the intervention programme is not yet well established, as there is the possibility of the procedure adding to distress at what can be an unsettling time for very young children. It is of course scientifically a very interesting question.

Testing the program is on its own is a useful contribution, as there is no established research base for it at present. There are references to the Thrive programme given in the proposal, numbers 9 to 11, which are links to reports. (Link to number nine was not working). The team has collected some pilot data on acceptability.
There were also different types of data proposed to be collected but why all these data will be collected and integrated and how these data will inform each other was not well argued. The qualitative data analyses were not well described.

Selected mark : 5 - Very good
The project's objectives, research questions and hypotheses are quite clearly presented and are based on a well-formulated and original project concept. The project will contribute to scientific innovation as well as generate new knowledge. The project is of very good quality, but has some minor weak points. Publications in recognised scientific journals in the field may be anticipated.

The project manager and project group

How would you rank the qualifications of the project manager and project group?

This criterion gives an indication of the qualifications of the project manager and project group. The project manager and project group will be assessed in relation to the following points:
* Project management
* Expertise and experience within the field of research
* Publication record
* Experience with national and international collaboration on projects
* Experience with supervision of students and younger researchers
* The degree to which the project manager and project group are part of a research environment that has the competence and resources needed to ensure the success of the project

The PI has the relevant expertise, a good publication record and has conducted several clinical trials.
The national team is also very good with relevant expertise.
The international collaborators are excellent and leaders in their fields.
The team have ample experience to supervise a research project of this kind.

Selected mark : 6 - Excellent
The project manager and/or research/project group is/are qualified at a high international level, has/have contacts within the foremost national and international research environments and will be able to play an important role in ensuring the success of the project.

Implementation plan and resource parameters

How well-suited are the implementation plan and resource parameters in relation to the project?

This criterion gives an indication of whether the plan for project implementation is satisfactory, and whether the planned use of resources in the project is well-suited for the tasks in the project, based on assessment of the following elements:
* Plans for project implementation, including breakdown into work packages/sub-projects, milestones and deliverables.
* Need for personnel resources, as listed in terms of work time distributed by work packages, sub-projects or milestones.
* Need for other resources (such as equipment, data collection, field work), distributed by work packages/sub-projects or milestones.

The assessment is not to be linked to any scientific risk.

The lack of existing evidence for the intervention makes it important to evaluate before implementation. The plan for evaluation set out in the research looks realistic. As there is a lack of evidence on the intervention, it has to represent a major piece of work and require considerable resources. The extent to which cost effectiveness will be evaluated is somewhat unclear.

Selected mark : A - Very good
The project plan and planned use of resources are very clearly described and are well-suited to the tasks in the project.

National cooperation

To what degree will the project promote national cooperation?

This criterion gives an indication of the extent to which the project will make use of national research expertise and help to promote national network-building.

The project will foster national cooperation, as the team already appear to be well integrated into policy related work.

Selected mark : A - Very good
The project will make comprehensive use of national research expertise and will contribute greatly to promoting national network-building.

International cooperation

How would you rank the international cooperation set out for the project?

This criterion gives an indication of the extent and quality of the international cooperation activities set out for the project.

The project will foster international cooperation, there is a very strong team with excellent international networking.

Selected mark : A - Very good
The international cooperation activities set out for the project have a wide scope and are of high quality.

Dissemination and communication of results

How would you rank the quality of the dissemination and communication plans?

This criterion gives an indication of the quality of the dissemination and communication plans for the project.
Dissemination and communication of results will be assessed in relation to the following points:
* Plans for scholarly publication, dissemination and other communication activities.
* Plans for popular science dissemination and communication activities vis-à-vis the general public as well as users of the project results, including planned use of channels and measures.
* Plans for ensuring that important users (in industry, community life and public administration) are incorporated into/take part in dissemination activities for the project.

When assessing dissemination and communication plans, importance should be attached to the level of detail provided and how realistic the plans are.

The proposal shows great strength in the way that it has aligned with policy and promotion of public health, and builds on many existing relationships. Dissemination strategies are mentioned relatively briefly, and seem appropriate.

Selected mark : A - Very good
The project’s dissemination and communication plans provide a thorough level of detail and are of high relevance.

User involvement

How would you rank the user involvement in the project?

This criterion gives an indication of the degree to which the project incorporates user perspectives and user involvement, or how well the absence of user involvement is explained. User involvement will be assessed based on the description of:
• Appropriate users for the project.
• Involvement of users in the planning of the project.
• Involvement of users in the implementation of the project.
• Involvement of users in the utilisation of project results.

User involvement is excellent. The childcare director has contributed to the pilot study and planning of the project and the national parents committee of Kindergartens is also an advisor to the project. User involvement seems a real strength of the proposal.

Selected mark : A - Very good
The user perspective and user involvement are very well incorporated in all phases of the project.

Benefits of the project

How would you rank the benefits of the project?

This criterion gives an indication of the significance and benefits that the results of the project will have for the users, the research field and society at large. The benefits of the project will be assessed on the basis of the following points:
• Benefits for the users and the research field.
• Benefits for society at large.
• Conditions that must be in place for implementing research findings in practice.

The projects has benefits to prevention of potential mental illness in young toddlers. As such the benefits are very high as it starts prevention earlier than any other study conducted so far in mental health interventions.
If successful, the programme will benefit children and families, which will lead to benefits of society at large, longer term. The conditions are already in place for implementation of the findings through into practice, and this is a major strength of the work.

Selected mark : A - Significant
The project has significant benefits.

Overall assessment of the referee/panel

How does the project rank in terms of the referee's/panel's overall assessment?

This criterion indicates the overall view of the referee/panel, based on the specific criteria which they have been asked to assess.

This is an excellently written project that will be very beneficial for a wide range of children.
The relevance of the study is clearly stated, the hypotheses clear, and the analyses well thought through and described.
It is unique in that the intervention has not been used in such small toddlers in Europe to my knowledge.
The improvement of child care has wide-ranging preventative beneficial effects to the mental health of children.
The team has the relevant expertise to conduct and analyse the study and the international cooperations are excellent.
The user involvement was a real strength of the proposal.
The projects has benefits to prevention of potential mental illness in young toddlers. As such the benefits are very high as it starts prevention earlier than any other study conducted so far in mental health interventions.
If successful, the programme will benefit children and families, which will lead to benefits of society at large, longer term. The conditions are already in place for implementation of the findings through into practice, and this is a major strength of the work.

There is little detail given on cost effectiveness in the main proposal. The need for the cortisol measurements was not made very clear as this may be a burden for the children.
Different types of data will be collected but it was not clear how all these data will be integrated and how these data will inform each. The qualitative data analyses were not well described.

Selected mark : 5 - Very good
A project of national and international interest. Publications in recognised journals may be anticipated. The researchers are very well recognised in their field.

Special points to consider

Special points to consider	Answer
Ethical perspectives	Yes
Environmental impact	Neutral
Recruitment of women	Significant
Gender balance in the project	Yes
Gender perspectives in the research	Positive
Internationalisation	Significant

Comments to special points to consider

On gender, the panel wondered how easy it will be to look at how male and female caregivers interact with the children, and what percentage of child care staff will be male.

Also there is the risk of increasing child distress if they are having difficulty settling in day care, and any additional distress that might be caused by cortisol sampling.
